# Supplementary material for: Structure and Formation Mechanism of Antimicrobial Peptides Temporin B- and L-Induced Tubular Membrane Protrusion
Source: Int J Mol Sci. 2021 Oct 13;22(20):11015. doi: 10.3390/ijms222011015 (PMC8537239; doi:10.3390/ijms222011015)
Supplement: Supplementary file 1 [file ijms-22-11015-s001.zip › ijms-1407381-supplementary.pdf]

Supplementary for

Structure and formation mechanism of antimicrobial peptides temporin B and L-induced tubular membrane protrusion

Shan Zhang, Ming Ma, Zhuang Shao, Jincheng Zhang, Lei Fu, Xiangyuan Li, Weihai Fang, and Lianghui Gao*

Table S1. Comparison of helicity, gyration radius (Rg) and solvent accessible surface area (SASA) of peptides at water-gas interface and membrane surface.

Peptide	Water-gas			Membrane		
	**helicity**	**Rg (Å)**	**SASA (nm^2)**	**helicity**	**Rg (Å)**	**SASA (nm^2)**
**TB**	62±6%	6.89±0.01	15.88±0.03	38±18%	7.25±0.52	16.12±0.68
**TL**	66±4%	7.48±0.08	17.69±0.02	64±7%	7.77±0.41	18.34±0.74

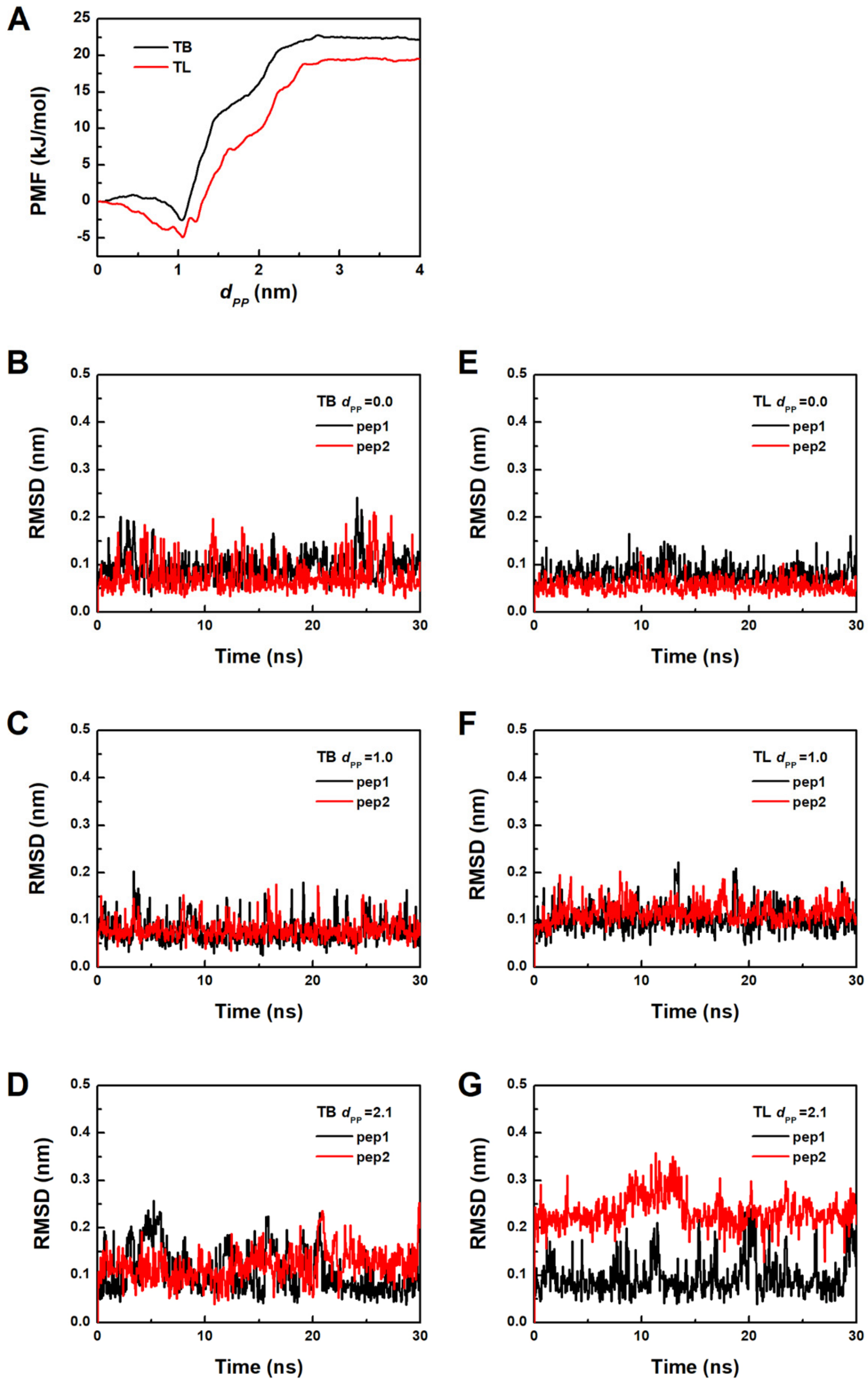

Figure S1. (A) Dimerization free energy of TB and TL at water-gas interface. Here, d_{PP} is the distance between the centroids of two peptides. (B-G) Root mean square deviation (RMSD) of TB and TL backbone at d_{PP} = 0.0, 1.0, and 2.1 nm. The black and red line represents peptide 1 and 2 in the dimer, respectively.

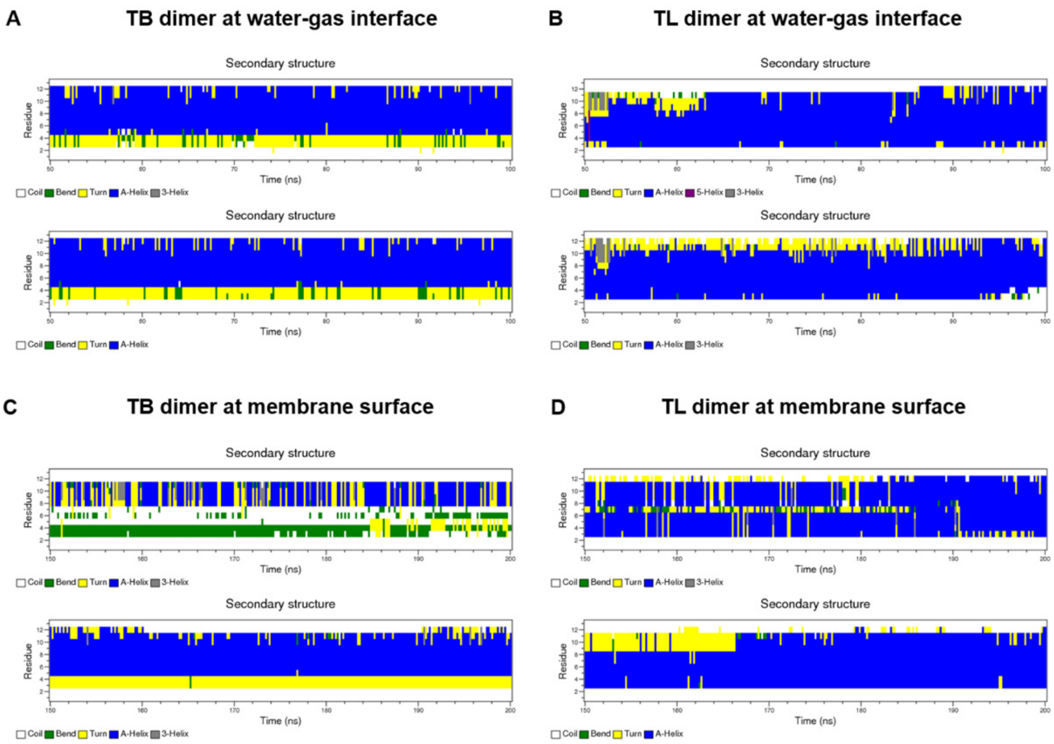

Figure S2. Secondary structure of TB (A,C) and TL (B,D) dimers at the water-gas interface and membrane surface.

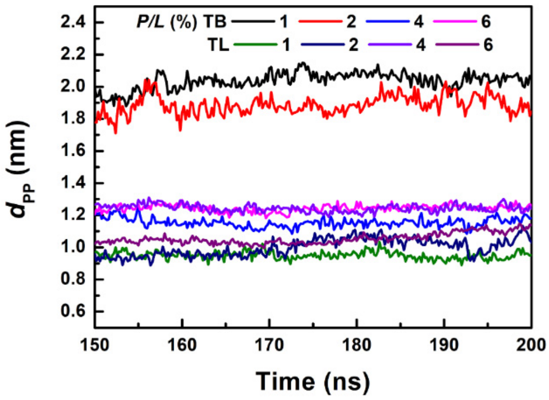

Figure S3. Distance between peptide centroids in each pair of dimer after TBs and TLs stably bound to membranes.

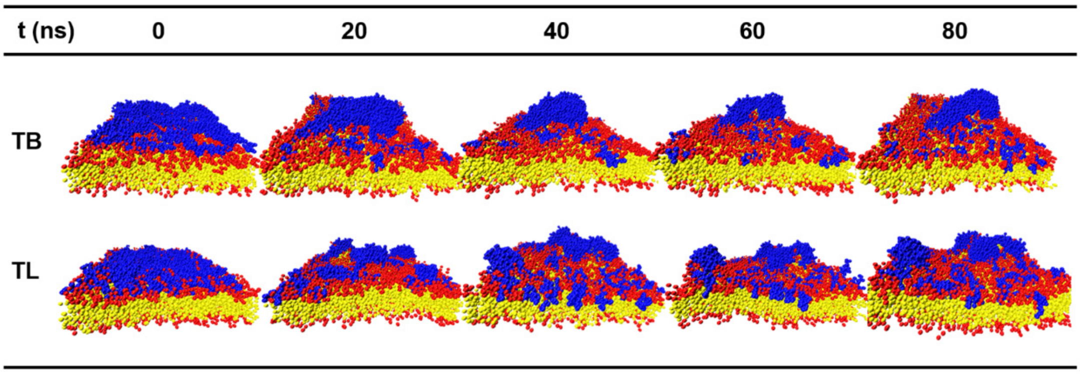

Figure S4. Snapshots of TB and TL binding onto lipid bilayer membrane at peptide concentration of *P*/*L* = 10% in the early stage.

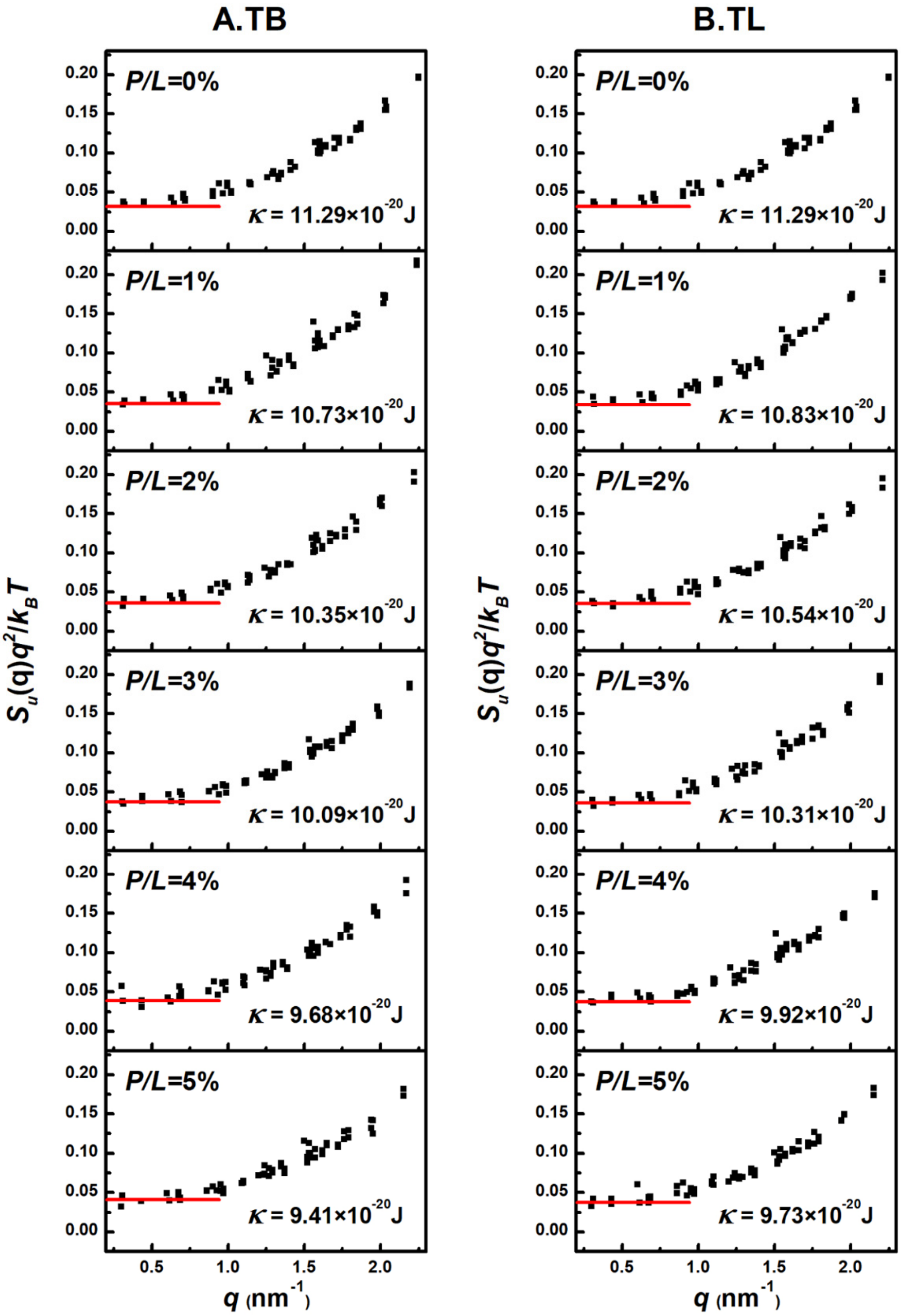

Figure S5. Spectrum of longitudinal lipid orientation fluctuations before and after TB and TL binding onto the membrane at different peptide concentrations. Converged values of κ were obtained from the plateau regions extending over at least four smallest wave vectors.
